# Examining the effects of pleasantness ratings on correct and false recognition in the DRM paradigm: accuracy, recollection and familiarity estimates

**DOI:** 10.3389/fpsyg.2024.1265291

**Published:** 2024-03-20

**Authors:** Alicia Alvarez-Martinez, Maria J. Sampedro-Vizcaya, Jose Fernandez-Rey

**Affiliations:** ^1^Department of Basic Psychology, Psychobiology and Methodology of Behavioral Science, Faculty of Psychology, University of Salamanca, Salamanca, Spain; ^2^Department of Social Psychology, Basic Psychology, and Methodology, University of Santiago de Compostela, Santiago de Compostela, Spain; ^3^Cognitive Processes and Behavior Research Group, Department of Social Psychology, Basic Psychology, and Methodology, University of Santiago de Compostela, Santiago de Compostela, Spain

**Keywords:** DRM paradigm, false recognition, distinctive encoding, item-specific processing, pleasantness rating, recollection, familiarity, mirror effect in recognition memory

## Abstract

Distinctive encoding usually increases correct recognition while also producing a reduction in false recognition. In the Deese-Roediger-McDermott (DRM) illusion this phenomenon, called the mirror effect, occurs when participants focus on unique features of each of the words in the study list. In previous studies, the pleasantness rating task, used to foster distinctive encoding, generated different patterns of results. The main aim of our research is to examine under what circumstances this task can produce the mirror effect in the DRM paradigm, based on evidence from recognition accuracy and subjective retrieval experience. In Experiment 1, a standard version (word pleasantness rating on a 5-point Likert-type scale) was used for comparison with two other encoding conditions: shallow processing (vowel identification) and a read-only control. The standard task, compared to the other conditions, increased correct recognition, but did not reduce false recognition, and this result may be affected by the number of lists presented for study. Therefore, in experiment 2, to minimize the possible effect of the so-called retention size, the number of studied lists was reduced. In addition, the standard version was compared with a supposedly more item-specific version (participants rated the pleasantness of words while thinking of a single reason for this), also including the read-only control condition. In both versions of the pleasantness rating task, more correct recognition is achieved compared to the control condition, with no differences between the two versions. In the false recognition observed here, only the specific pleasantness rating task achieved a reduction relative to the control condition. On the other hand, the subjective retrieval experience accompanied correct and false recognition in the various study conditions. Although the standard pleasantness rating task has been considered to perform item-specific processing, our results challenge that claim. Furthermore, we propose a possible boundary condition of the standard task for the reduction of false recognition in the DRM paradigm.

## 1 Introduction

A common type of memory distortion is false recognition, in which individuals erroneously claim that a specific item or event was previously encountered or experienced. This plays an important role in understanding the potential fallibility of human memory, showing that our memories are not always accurate and can be susceptible to errors and distortions. It also has significant implications in legal and forensic contexts, particularly in eyewitness testimony or when individuals falsely recognize a person or object, leading to wrongful accusations or indeed convictions (Schacter, 1995). Hence, the study of false recognition contributes to improving our appreciation of the accuracy and reliability of memory and thus minimizing the possible negative consequences of distortions that may arise (e.g., Dodson et al., 2000). However, one question that remains open is exactly how we can avoid, or at least minimize, false recognition. In this paper we focus on the effects of distinctive encoding as a technique for reducing false recognition, using a standard laboratory paradigm for the creation of false memories.

One of the best-known experimental paradigms for the study of false memories in the laboratory was initially proposed by Deese (1959), later adapted and extended by Roediger and McDermott (1995), and is currently called the Deese-Roediger-McDermott paradigm, or DRM. In general, the approach involves the study of lists of words with a strong semantic association, where each list converges with a non-studied word, referred to as the *critical lure*. For example, the list may contain words such as *thief, bars, cell, detention, prisoner*, etc., which are implicitly associated with the critical lure *jail*. Results of this task indicate very consistently a tendency to falsely recall/recognize the critical lures, and frequently, with a high degree of confidence, of having perceived these in the study phase. This effect, also called the *DRM illusion*, has been shown to be robust under different experimental conditions (for a review, see Gallo, 2010; Roediger and Gallo, 2022).

Two main theories have been proposed to explain the DRM illusion: the *Fuzzy-trace theory* (FTT; *cf*., Reyna and Brainerd, 1995; Brainerd and Reyna, 2002) and *Activation-monitoring theory* (AMT; *cf*., Roediger et al., 2001a,b). These approaches concur in claiming that there are two processes that interact in memory tasks, one related to error-inflating and the other to error-editing. These processes would work together to improve performance in true memories, while working in opposite directions in false memories: inflation processes would serve to increase false memories whereas editing processes would decrease them (Arndt and Gould, 2006). Specifically, FTT assumes that two types of representations or traces are encoded in memory during the study of DRM lists: a specific representation (*verbatim*), based on the perceptual details and shallow structures of words, and a generic representation (*gist*), based on the general meanings of words (Reyna and Brainerd, 1995; Brainerd and Reyna, 2002). According to this theory, false memories, unlike true ones, are seen to be supported by processes based on *familiarity*. Critical lures, being semantically associated with the words in the list, share meaning information with the gist representation generated in the study phase; the more they share, the more of a sense of familiarity they generate, and therefore the greater is the probability of false memories arising. The reduction of false memories is favored by enhancing the verbatim representations, thus increasing the specific details of the items studied to reduce familiarity with the critical items. For its part, the AMT argues that false memories are produced by the interaction of activation processes during encoding and monitoring processes during retrieval (Roediger et al., 2001a,b). This theory, based on the spreading activation model of Collins and Loftus (1975), proposes that, in the study of DRM lists, the presentation of each associated word of the list produces the activation of its corresponding node, propagating automatically through the semantic network and leading to the activation of the critical lure in a recurrent manner, given its proximity in the network. The level of activation is determined by the associative strength between the words in the list and the critical word; thus, the higher the level of activation of critical lures, the more likely are false memories. For the reduction of these, monitoring processes are launched, which seek to identify the origin or source of the activation using different types of information (Gallo, 2006). According to this theory, false memory occurs when the origin of the activation of the critical lures (internal associative activation) is erroneously attributed to the study phase (external associative activation), resulting in a source-monitoring error.

One of the justifications for the claim that the retrieval of critical lures DRM can be characterized as false memory is based on the fact that the associated subjective experience is comparable to that which accompanies the retrieval of the studied words. Such evidence is extremely important because it indicates that DRM false recognition is not due to a guessing strategy or a change of response criterion (Dewhurst and Farrand, 2004; Schacter et al., 2021). To assess the subjective experience associated with recognition, the remember/know procedure developed by Tulving (1985) is commonly used, in which participants are asked to report their state of consciousness associated with each affirmative response. When they can recall specific details about the occurrence of an item, they are asked to respond with a *Remember* judgement, and when they cannot recall specific details, but recognize the item because it is familiar to them, they should respond with a *Know* judgement. Remember responses are considered to correspond to the *recollection* process, which involves autonoetic awareness, whereas Know responses correspond to the process of familiarity, which involves a noetic awareness (Gardiner, 2001). In line with dual-process theories (e.g., Yonelinas, 1999), familiarity-based recognition is fast and automatic; by contrast recollection-based recognition is slow and more demanding, since it is the one that allows for recalling the details of the episode in question. Currently, there is no general agreement as to the characterization of subjective experience in false recognition (Schacter et al., 2021). However, we have found some approaches from FTT and AMT. According to FTT, Remember responses, based on recollection, are supported by verbatim representation, since specific information about the context of the presentation of the items is requested; while Know responses, based on familiarity, are supported by gist representations. Since this theory conceives that false memories are only produced by familiarity processes (gist representation), the higher proportion of Remember responses to critical lures cannot be explained in terms of verbatim representation, but is due to a strong sense of familiarity that is mistaken for a sense of recollection (phantom recollection; Brainerd et al., 2001). For AMT, on the other hand, false memories may be supported by recollection processes. The subjective experience in false recognition, mostly of Remember responses, could be linked to the illusory retrieval of associative, contextual, and perceptual details about the encoding of studied words. That is, specific details that are (illusorily) recalled from critical lures could be due to the erroneous attribution of encoded information about studied words (Roediger et al., 2004; Lampinen et al., 2005).

Of the different techniques used to reduce the DRM illusion, one of the most effective is to foster the distinctive encoding of items in the study phase. The notion of distinctiveness has allowed for an enriching of the levels-of-processing approach (Craik and Lockhart, 1972), in that the recall of an item will be better when its encoding results in a memory trace with unique or distinctive properties in relation to the other items in the list. Thus, deep levels of processing might encourage the distinctiveness of the items studied. On the other hand, it is also known that, during the encoding process, organizing information around similarities or grouping items into higher categories facilitates retrieval (Bower et al., 1969; Flores et al., 2017). Thus, with the aim of integrating the levels-of-processing approach (by means of the incorporation of the idea of distinctiveness) and the organization-based approach (which emphasizes similarity), the relational-distinctive theoretical framework was designed, this being based on the distinction between relational and individual or item-specific information (Einstein and Hunt, 1980). This framework posits that the retrieval of an item depends on both the relationships between the other items in the episode and the distinctive characteristics of that item. Hence, depending on the demands of the task, we can perform relational processing, encoding items on the basis of their similarities, or item-specific processing, encoding them on the basis of their individual and unique attributes.

There is considerable debate in the literature as to whether the benefits of distinctive encoding in the DRM paradigm can be attributed to encoding or retrieval processes (Huff et al., 2015), with two explanatory approaches having garnered the most evidence: the *distinctiveness heuristic* approach (Dodson and Schacter, 2001; Schacter et al., 2001), in relation to monitoring strategies in retrieval, and the *impoverished relational encoding* explanation (Arndt and Reder, 2003; Mccabe et al., 2004), in relation to the relational-distinctive framework. The former approach refers to the fact that, having performed distinctive encoding, a diagnostic decision-making strategy is adopted in the memory test. That is, when studying DRM lists through performing distinctive processing (e.g., associating words with an image), if at the time of retrieval distinctive details of the items (the associated images) are recalled, evidence is obtained that they were studied; conversely, the absence of these details indicates that they were not studied. When critical lures appear in the test, since they were not studied, the information that one would expect (the image) is not recalled; therefore, this absence is what facilitates their rejection (Schacter et al., 2001). By turn, impoverished relational encoding proposes that, given that the nature of DRM lists favors relational processing due to the semantic relationships therein, item-specific processing should be increased to encourage discrimination. Thus, distinctive encoding would encode less information about the similarities between the words in the (relational) list, but more individual information. In the DRM paradigm, this would result in reduced activation of critical lures, according to AMT, or the altered thematic coherence of the list, according to FTT, implying greater discrimination between studied and critical words in the memory test (Mccabe et al., 2004).

Of the methodologies developed to separate the effects of distinctiveness in recognition due to encoding processes from those due to retrieval processes, one of the most widely accepted at the moment is based on the *signal detection theory* (SDT; Gunter et al., 2007; Huff and Bodner, 2013; Bodner et al., 2017; Huff and Aschenbrenner, 2018). Signal detection analyses attempt to separate participants’ ability to discriminate between studied versus non-studied items from their response bias, or the tendency to report in the memory test that an item was studied. For discriminability, we calculate the *d’* index, which estimates the amount of information in memory that has been encoded in a condition, and for response bias, we calculate the lambda (*λ*) index, which reflects whether responses are more conservative (involving more monitoring) or more liberal (less monitoring). Both indexes can be calculated for words of the DRM list as well as for critical lures. Applying this methodology, Gunter et al. (2007) found that performing a distinctive encoding, such as an anagram generation task, compared to a read-only control encoding, led to a reduction in false recognition. Signal detection indexes showed that this was a result of both encoding processes, due to less information encoded on the critical lures, and retrieval processes, due to increased monitoring in the memory test. This and other studies (e.g., Hanczakowski and Mazzoni, 2011) suggest that both types of process contribute to the reduction of false memories in the DRM paradigm when distinctive encoding is performed. That is, the impoverished relational encoding and distinctiveness heuristic explanations do not appear to be mutually exclusive, but rather complementary.

On the other hand, Huff and colleagues (Huff and Bodner, 2013, 2019; Huff et al., 2015) have argued that the reductions in the DRM illusion obtained in previous studies were not due to the performance of a distinctive processing task *per se*, but depended on the extent to which the specifics of those tasks induced item-specific versus relational processing. Therefore, in order to compare the effects of both types of processing on recognition and free recall, they used different versions of tasks, ones which had presumably achieved a reduction of DRM illusion, such as anagram generation and pleasantness rating. Although participants encoded the words with the same task, the instructions they were given induced a search for similarities (relational version) or differences (item-specific version). Among the results observed, both item-specific processing and relational processing promoted correct recognition compared to the control group (read-only condition); however, only item-specific processing was able to reduce false recognition (Huff and Bodner, 2013). Results in free recall were similar, except that relational processing reduced false recall compared to the reading group, although less so than item-specific processing (Huff and Bodner, 2019). Furthermore, signal detection analyses supported the explanation provided by the distinctiveness heuristic and impoverished relational encoding approaches, since in the conditions where the DRM illusion was reduced, the information encoded in memory of critical lures decreased, whereas monitoring in the memory test increased. In sum, it was observed that performing item-specific processing facilitated the *mirror effect* pattern of results (increase in correct memory and reduction in false memory; Glanzer and Adams, 1990) in both free recall and recognition; however, when performing relational processing, the pattern only appeared in free recall, and to a lesser degree.

Turning to the effects of the pleasantness rating task on DRM illusion, several considerations should be taken into account. First, different versions of the task and different comparison conditions have been used, leading to different patterns of results. In free recall, with the standard version, where the pleasantness of each item is rated on a scale of 1–5, a more is less pattern was obtained (increased correct recall, but also increased false recall) compared to a shallow encoding (Toglia, 1999); however, with the same version of the task, a mirror effect pattern was also obtained compared to a reading control group (Hunt et al., 2011). Second, regarding item-specific vs. relational processing, Huff and Bodner (2013, 2019) did not use the standard task in their research, but designed other versions of it. With the item-specific version (requiring participants to think of a single reason why the studied words were pleasant or unpleasant), they observed a mirror effect in both free recall and recognition, but not with the relational version (asking them to rate the pleasantness of each word in comparison to the word previously presented in each list, on a Likert-type scale from 1 to 7). Third, Huff and Bodner’s results contrast with those obtained using the standard task. Although the standard task is considered to be an item-specific processing task, in that it encourages attention to individual information (Einstein and Hunt, 1980; Hunt, 2013), the mirror effect pattern that might be expected was only observed in free recall (Hunt et al., 2011), not in recognition (Gretz and Huff, 2020). These discrepancies, caused by different methodological variations, highlight the lack of a satisfactory explanation for the effects of the pleasantness rating task in the DRM paradigm.

The main objective of our current study is to explore the effects of the pleasantness rating task, in comparison with other types of encoding, on the DRM paradigm. More specifically, the aim is to observe whether there are differences in correct and false recognition as a function of the encoding performed, thus testing whether the pleasantness rating produces the mirror effect in recognition memory. Furthermore, we aim to characterize the subjective retrieval experience associated with correct and false recognition as a function of the type of encoding, evaluating the recollection and familiarity processes in recognition memory. Finally, we are interested in comparing the effects of different study conditions on encoding and retrieval processes separately, on the basis of indexes derived from the theory of signal detection. In Experiment 1, only one version of the pleasantness rating task was used, while in Experiment 2, two versions were used.

## 2 Experiment 1

A standard version of the pleasantness rating task was used to make a comparison with two other encoding conditions (shallow processing and control). We expected to find that the DRM list study would generate a high rate of false recognition of critical lures in the participants, regardless of the type of encoding performed (classic DRM effect). In addition, the pleasantness rating, if it involves item-specific processing, would produce higher correct recognition and lower false recognition than the control condition (mirror effect). In the shallow processing condition, we would expect a lower rate of correct recognition, but a lower or similar rate of false recognition as in the deep/distinctive processing condition (pleasantness rating), since less information about the studied words is encoded. Retrieval after distinctive encoding (pleasantness rating) would be guided by recollection rather than familiarity processes, in that more specific information (distinctive details) about the studied words is available, compared to shallow encoding, in which there is limited information about them. Finally, the reduction in the DRM illusion when performing distinctive encoding would be due to both encoding mechanisms, which would reduce the information encoded in memory of the critical lures, and retrieval mechanisms, which would increase monitoring in the recognition test.

### 2.1 Materials and methods

#### 2.1.1 Participants

A total of 119 Psychology students from the University of Santiago de Compostela, all native Spanish speakers,^1^ participated voluntarily and were awarded with a course credit. Data from five participants were eliminated due to extreme scores, leaving 114 participants for analysis (97 women; *M*_age_ = 19.44, *SD* = 1.74). The sample was divided into three groups according to the type of encoding: Control (*n* = 40), Standard (*n* = 39) and Shallow (*n* = 35). Sensitivity analysis conducted using GPower 3 (Faul et al., 2007) indicated that the sample size was sufficiently large (0.80) to detect medium-sized effects (*f* = 0.29) for the main effect of encoding type. The study was approved by the bioethics committee of the University of Santiago de Compostela.

#### 2.1.2 Design

The study presents two independent variables: A between-subject variable of Encoding Type (reading control condition, identification of the letter “E” or shallow condition and standard pleasantness rating), where participants were randomly assigned to each condition, and a within-subject variable of Word Type (studied, critical, unrelated distractor and unrelated distractor-critical). In addition, three dependent variables were measured: Recognition, Remember Responses (R) and Know Responses (K), percentage of “YES,” “R” or “K” responses to the different words in the recognition test.

#### 2.1.3 Materials

Thirty-two DRM lists of Spanish words selected from a previous study were used (c.f., Beato and Díez, 2011). Although these lists were composed of 6 words associated with 3 critical lures, in the present study only one critical lure was used, namely the one that provided the highest rate of false recognition. These lists provided high levels of false recognition and had a minimum backward associative strength (BAS) value of 0.03 and a maximum of 0.98 (Beato and Cadavid, 2016). Thirty-two were selected to keep the task demand equivalent to previous studies on distinctive encoding (Huff and Bodner, 2013), given that DRM BAS lists in English typically contain 12 associated words, while the lists available in Spanish contain 6. The order of the words within each list was fixed, because they are ordered in decreasing order of associative strength with the corresponding critical lure. An extra list was also used as a practice example. Materials for our experiments are provided in our OSF project: https://osf.io/rzyjc/.

The 32 lists were divided into two counterbalanced sets of 16 lists each, matched in terms of BAS and false recognition rate, where one set was studied and the other set functioned as a control set. In the construction of each set, lists with the same subject matter and repetition of words were avoided. Thus, with this arrangement, the 4 types of words used in the test were obtained: studied, critical, unrelated distractor, and unrelated critical distractor. The distractor task involved 60 solved arithmetic operations, these including additions and subtractions. Since it was a verification task, only half of these were correct (e.g., 11–2–5 = 2; 10 + 4 + 8 = 22).

The recognition test, with 96 words, included 48 studied, 16 critical, 24 distractor and 8 critical distractors (48 studied/48 non-studied), similar to the procedure used by Roediger and McDermott (1995). The studied words corresponded to positions 2, 4 and 6 in each of the 16 studied lists. The non-studied words comprised the 16 corresponding critical lures from the studied lists and the remaining 32 words were drawn from 8 lists of the non-studied set, using positions 2, 4 and 6 as unrelated distractors and the associated critical lures as non-related critical distractors. To ensure that all lists were subjected to all conditions, 4 test versions were constructed for each set. Two versions used the first 8 control lists of the non-studied set (each with a different order of word presentation) and the other two versions used the last 8 lists. The creation of different word presentation orders was undertaken to prevent the specific order from affecting the results. The order of word presentation was pseudo-randomized using a procedure similar to that in Graham (2007), where critical lures were separated by at least two items and words from the same list were also separated by at least two other words from different lists.

#### 2.1.4 Procedure

All participants were tested individually with an experimenter present. Participants performed the presented tasks on a computer with the software PsychoPy3 (Peirce et al., 2019). A QWERTY keyboard was used to collect responses with the number keys (1, 2, 3, 4 and 5) and with the keys “z,” “x,” “n” and “m,” these with stickers added: “REC” (Response R, English REMEMBER), “SÍ” (English Yes), “NO,” “SAB” (Response K, English KNOW), respectively. After signing an informed consent form, participants reported their age and gender, all data being treated anonymously.

In each study condition specific instructions were presented at the beginning of the study phase. In the read-only condition participants were asked to pay attention to each of the words and to try to remember them. In the standard condition, participants were asked to rate the perceived pleasantness of each word on a scale of 1–5, with 1 being very unpleasant and 5 being very pleasant, using the number buttons on the keyboard. Participants in the shallow condition had to decide whether each word contained any letter “E,” answering using the buttons marked YES or NO on the keypad. The word lists were presented visually on a computer screen. Participants studied 16 lists presented in random order, with each word remaining for 2000 ms individually and with an inter-stimulus interval of 500 ms. Prior to the study phase, participants were given a practice list to practice the assigned study condition and the experimenter provided feedback to them if the strategy was not applied correctly.

At the end of the study phase, participants performed a distractor task, in which they had to review a series of already-solved arithmetic operations and decide whether the result was correct or not. Over the course of 2 min, the arithmetic operations were presented one at a time and each participant gave their answer with the YES or NO buttons on the keyboard. Finally, the recognition test was administered, in which it was indicated that they would be presented with words which were either previously studied words or not. They were asked to press the YES button if they thought it was a “studied” word, and NO button if they thought the word presented was “new.” In addition, if they decided that the word is “studied” they had to make a Remember/Know judgement. Following Rajaram (1993) they were instructed to respond with “remember” when they were able to recall some specific detail of the word’s presentation (some feature, what they were thinking, some picture...) and respond with “know” if they could not recall any specific detail of its presentation, but recognize it through familiarity; for this they had to respond with the REC or SAB keys on the keyboard. A trial run with the practice list was given beforehand to familiarize participants with the task and to resolve any doubts. Finally, they were thanked for their participation.

Prior to undertaking the research, a pilot study was carried out to assess the feasibility and duration of the study, as well as to identify possible errors.

### 2.2 Results

An alpha level of 0.05 was set for all analyses. Effect size estimates were provided for all significant comparisons using the partial-eta-squared coefficient (*η*_p_^2^) for the analysis of variance (ANOVA), and Bonferroni correction was applied for *post hoc* analyses.

#### 2.2.1 Preliminary data analysis

Signal detection: Following Gunter et al. (2007), indexes of discriminability (*d’*) and response bias (*λ*, lambda) were calculated for list items and critical items. The *d’* index of list items was calculated as the difference between the z-score^2^ of the hit rate for the items studied minus the z-score of the false alarm rate for the distractor items. The *d’* index of critical items was calculated based on the difference between the false alarm rate z-score of the critical items (treated as hits) minus the false alarm rate z-score for the critical distractor items. In addition, the *lambda* of the list items was calculated by taking the z-score of 1 minus the false alarm rate for the distractor items, and the *lambda* of critical items was calculated by taking the z-score of 1 minus the false alarm rate for the critical distractor items. False alarm rates of 0 and hit rates of 1 were adjusted using the 1/2n correction of Macmillan and Creelman (1991). Table 1 sets out the mean proportion of “Yes” responses and mean signal-detection indexes on the recognition test as a function of item type for the encoding conditions.

**Table 1:** Mean (*SD*) proportion of “yes” responses and signal-detection indexes on the recognition test as a function of encoding condition and item type for Experiment 1.

Encoding group/item type/index	Control	Standard	Shallow
*n*	40	39	35
List items	0.68 (0.12)	0.87 (0.10)	0.51 (0.14)
Unrelated distractors	0.04 (0.05)	0.05 (0.07)	0.12 (0.09)
List-items *d’*	2.25 (0.41)	3.08 (0.57)	1.31 (0.57)
List-items *λ*	1.76 (0.32)	1.73 (0.42)	1.29 (0.48)
Critical lures	0.46 (0.23)	0.44 (0.19)	0.41 (0.14)
Unrelated-critical distractors	0.11 (0.14)	0.09 (0.11)	0.20 (0.17)
Critical-lures *d’*	1.07 (0.78)	1.04 (0.64)	0.63 (0.50)
Critical-lures *λ*	1.19 (0.46)	1.23 (0.38)	0.88 (0.54)

**Figure 1:**
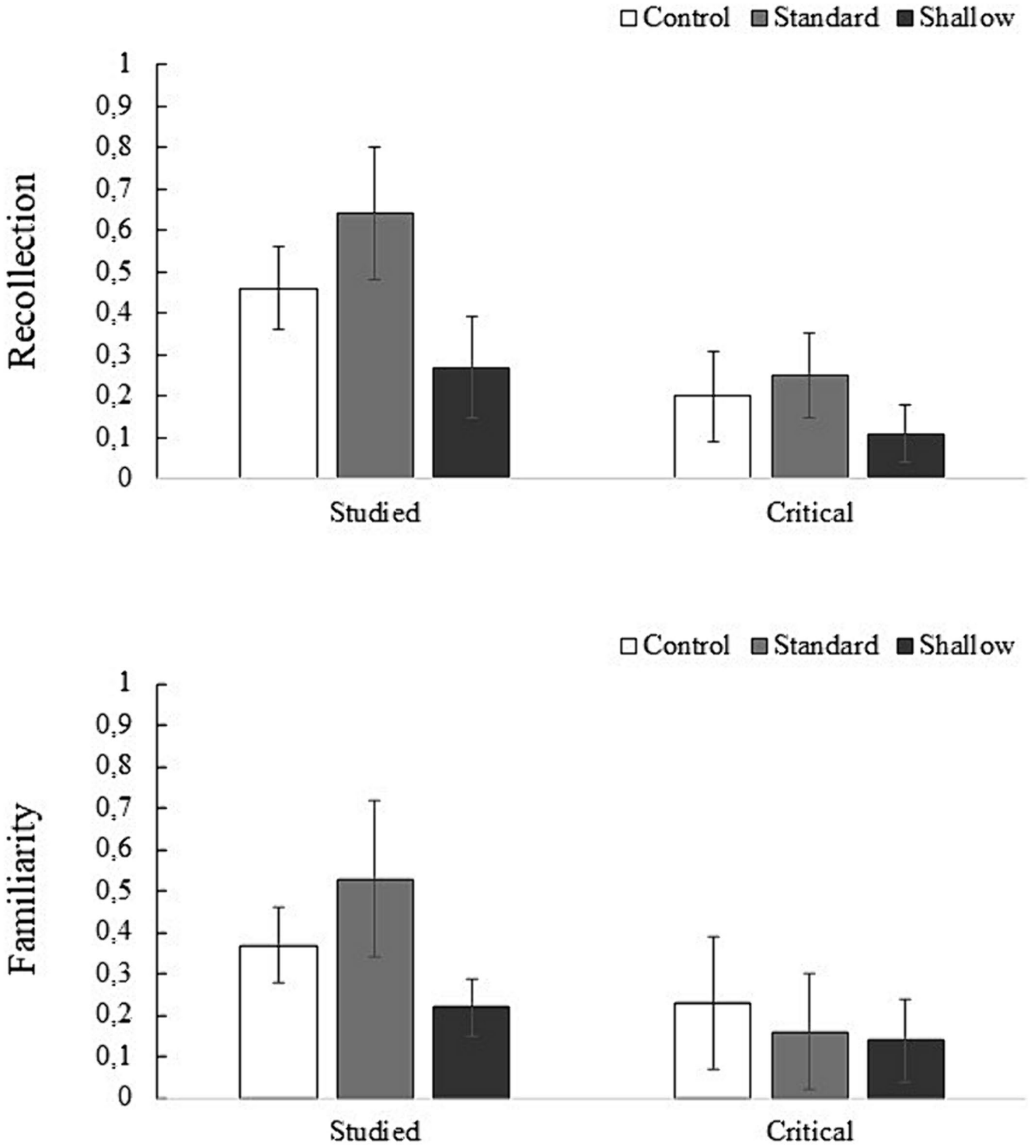
Mean (bars = ± 95% CI) estimates of recollection and familiarity for studied words and critical lures by encoding type for Experiment 1.

Subjective retrieval experience: Data for the raw R and K responses are given in our OSF project: https://osf.io/rzyjc/. Since raw “Know” responses underestimate the familiarity process, estimates of the processes of recollection and familiarity were carried out with the independence procedure for R/K responses developed by Yonelinas et al. (1998). For both list items and critical lures (understood as “old” items), the recollection index was estimated as: [(Rold - Rnew)/(1 - Rnew)], where distractor words function as “new” items. For familiarity we used the *d’* derived from the familiarity estimate, [Familiarity old = Kold / (1 - Rold) - Familiarity new = Knew/(1 - Rnew)]. Figure 1 shows the recollection and familiarity estimates for the studied and critical items for each type of encoding.

#### 2.2.2 Correct recognition

##### 2.2.2.1 Accuracy

The analysis of variance (ANOVA) performed on the recognition of the studied words showed a significant effect of encoding type, *F*(2, 111) = 91.37, *p* < 0.001, *η*_p_^2^ = 0.62. *Post-hoc* comparisons indicated that more words were recognized in the standard condition than in the control condition (0.89 vs. 0.68), *t*(77) = 7.55, *p* < 0.001, as well as in the shallow condition (0.89 vs. 0.51), *t*(72) = 13.47, *p* < 0.001. Also, more words were recognized in the control condition than in the shallow condition (0.68 vs. 0.51), *t*(73) = 6.21, *p* < 0.001. We also conducted an ANOVA on the *d’*_list_ index, which reflects memory information encoded about studied words. There was a significant effect of the type of encoding, *F*(2, 111) = 107.66, *p* < 0.001, *η*_p_^2^ = 0.66, with standard condition presenting the greatest *d’*_list_ in comparison to the control (3.07 vs. 2.26), *t*(77) = 7.05, *p* < 0.001, and shallow (3.07 vs.1.31), *t*(72) = 14.67, *p* < 0.001; additionally, the control presents greater *d’*_list_ than the shallow condition (2.26 vs. 1.31), *t*(73) = 7.05, *p* < 0.001. The ANOVA performed on the *λ*_list_ index, which reflects memory monitoring of studied words, showed a significant effect of encoding type, *F*(2, 111) = 14.89, *p* < 0.001, *η*_p_^2^ = 0.21. It was observed that the standard and control conditions were greater for *λ*_list_ than the shallow condition (1.74 vs. 1.29), *t*(72) = 4.61, *p* < 0.001, and (1.76 vs. 1.29), *t*(73) = 4.92, *p* < 0.001, respectively. By contrast, monitoring of studied words was similar in the standard and control conditions (1.74 vs. 1.76), *t*(77) = 0.28, *p* = 1.000.

##### 2.2.2.2 Subjective retrieval experience

The ANOVA performed on the recollection estimate of the studied words showed a significant effect of encoding type, *F*(2, 111) = 32.14, *p* < 0.001, *η*_p_^2^ = 0.37. Standard encoding showed higher recollection than in the control (0.64 vs. 0.46), *t*(77) = 3.98, *p* < 0.001, and shallow conditions (0.64 vs. 0.27), *t*(72) = 8.02, *p* < 0.001; in addition, the control condition showed more recollection than the shallow one (0.46 vs. 0.27), *t*(73) = 4.19, *p* < 0.001. A final ANOVA, performed on the familiarity estimate of the studied words, showed an effect of encoding type, *F*(2, 111) = 22.32, *p* < 0.001, *η*_p_^2^ = 0.29. Standard encoding yielded higher scores than in the control and shallow conditions (0.53 vs. 0.37), *t*(77) = 3.54, *p* = 0.002, and (0.53 vs. 0.22), *t*(72) = 6.67, *p* < 0.001, respectively. Again, the control condition showed more familiarity than the shallow condition (0.37 vs. 0.22), *t*(73) = 3.27, *p* = 0.004.

#### 2.2.3 False recognition

##### 2.2.3.1 Accuracy

The ANOVA performed on the recognition of the critical lures showed no significant effect of encoding type, *F*(2, 111) = 0.66, *p* = 0.517. The same ANOVA was performed on memory information encoded about critical lures, *d’*_critical_, and a significant effect was found of the type of encoding, *F*(2, 111) = 5.26, *p* = 0.007, *η*_p_^2^ = 0.09. No difference was found between standard and control conditions (1.04 vs. 1.07), *t*(77) = 0.21, *p* = 1.000; however, both conditions present a greater *d’*_critical_ than the shallow condition (1.04 vs. 0.63), *t*(72) = 2.72, *p* = 0.023, and (1.07 vs. 0.63), *t*(73) = 2.94, *p* = 0.012, respectively. The same analysis was also conducted for memory monitoring of the critical lures, *λ*_critical_, and showed a significant effect of the type of encoding, *F*(2, 111) = 5.98, *p* = 0.003, *η*_p_^2^ = 0.10. It was observed that standard and control conditions showed greater *λ*_critical_ than the shallow condition (1.23 vs. 0.88), *t*(72) = 3.17, *p* = 0.006, and (1.19 vs. 0.88), *t*(73) = 2.86, *p* = 0.015, respectively; however, monitoring was similar in the standard and control conditions (1.23 vs. 1.19), *t*(77) = 0.34, *p* = 1.000.

##### 2.2.3.2 Subjective retrieval experience

In the recollection estimate of the critical lures there was a significant effect of the type of encoding, *F*(2, 111) = 6.35, *p* = 0.002, *η*_p_^2^ = 0.10. The control encoding was not significantly different from either the standard or shallow conditions (0.20 vs. 0.25), *t*(77) = 1.42, *p* = 0.478, and (0.20 vs. 0.11), *t*(73) = 2.19, *p* = 0.092. However, the standard condition showed greater recollection than the shallow one (0.25 vs. 0.11), *t*(72) = 3.55, *p* = 0.002. The same ANOVA on the familiarity estimate of the critical lures showed no effect of encoding type, *F*(2, 111) = 2.18, *p* = 0.118.

### 2.3 Discussion

The pleasantness rating task (standard version) increased correct recognition compared to the other two encoding conditions (shallow processing and control). This result is consistent with previous studies (Gallo et al., 2008; Gretz and Huff, 2020). After performing deep/distinctive encoding, working with information on an item’s meaning, the information encoded about the studied words increases, making it more likely that a better performance will be obtained. Signal detection analyses showed how in correct recognition there was more encoded information (*d’*_list_) in the standard condition compared to the other two encodings, a result similar to that obtained by Huff and Bodner (2013). As far as monitoring (*lambda*_list_) is concerned, no differences were observed between standard and the control condition, contrary to what we expected. The standard task increased correct recognition relative to reading, but signal detection analyses showed that only increased memory information, not memory monitoring, contributed to this pattern. However, the standard and control conditions seem to trigger more monitoring mechanisms than shallow encoding. When poor semantic processing is performed on the items studied, shallow encoding fails to encode sufficient information; moreover, response bias tends to be more liberal, as less is monitored, all of which is reflected in lower performance (Gallo et al., 2008).

Turning to false recognition, the results of this experiment show no differences between encoding types in the recognition of critical lures. On the one hand, no differences are observed between shallow encoding and the pleasantness rating. This is not surprising because, although a more is less pattern is to be expected in free recall (Toglia, 1999; Hunt et al., 2011), no significant differences have been found in recognition (Beato et al., 2012; Gretz and Huff, 2020). Signal detection analyses showed significant differences with respect to the information encoded on the critical lures (*d’*_critical_), with shallow encoding presenting less memory information; furthermore, there are differences in terms of monitoring (*lambda*_critical_), since a laxer criterion is adopted in the responses. This is explained by the fact that, through attending to perceptual characteristics, no information on the meaning of the studied words is encoded, which does not favor the activation of the critical words, according to the AMT, or of thematic coherence, according to the FTT. Shallow encoding, in a way, induces impoverished relational processing, with less information available about the critical lures; however, the monitoring is weaker in recognition, which makes it more susceptible to errors (Gallo et al., 2008). In the end, deep/distinctive encoding obtains similar results to shallow encoding, but by means of different procedures. Despite encoding more information about the critical lures, a more conservative approach in responses is adopted in distinctive encoding.

On the other hand, there is also no reduction in false recognition in the pleasantness rating task compared to the control condition. Unlike the mirror effect found in free recall (Hunt et al., 2011) and in recognition with item-specific versions (Huff and Bodner, 2013), this standard pleasantness rating task does not manage to reduce false recognition compared to a reading condition, a result similar to that found by Gretz and Huff (2020). Furthermore, signal detection analyses show no differences between standard and the control condition, either in the amount of information encoded on critical lures (*d’*_critical_) or in the monitoring performed on critical items (*lambda*_critical_). This contrasts with what Huff and Bodner (2013, 2019) found: when comparing item-specific encoding and one of reading, they observed a reduction in the amount of information encoded on the critical lures (impoverished relational encoding) and an increase in monitoring (distinctiveness heuristic).

As far as the subjective retrieval experience is concerned, differences are observed according to the type of encoding. As expected, in the deep encoding condition (pleasantness rating), a higher estimate of recollection is obtained in both correct and false recognition compared to shallow encoding. This typical effect of processing levels has been observed in several studies (e.g., Gardiner and Parkin, 1990); thus, it appears that more specific details are recalled in conditions where more information about the studied words has been encoded. However, in false recognition, what occurs is an illusory retrieval accentuated in deep/distinctive encoding. More recollection to critical lures may stem from the higher encoded information about the studied words, which either misattributes them to the critical lures, as proposed by AMT, or generates an extreme sense of familiarity that leads to phantom recollection, according to FTT.

In this experiment, the standard version of the pleasantness rating task in the DRM paradigm did not yield the mirror effect in recognition because it did not induce distinctive/item-specific processing sufficiently under these study conditions. However, it has sometimes been considered as a task that does induce item-specific processing, this by encouraging attention to individual information (Einstein and Hunt, 1980; Hunt, 2013); indeed, the mirror effect was found with this task in free recall tests (Hunt et al., 2011). It is true that DRM lists induce relational processing due to the semantic relations presented by the words, and therefore in order to obtain better accuracy and a reduction of the DRM illusion, item-specific processing should be encouraged. This is especially relevant in recognition tests, where individual item information is key to discrimination. Therefore, given the inconsistent results regarding the effects of the pleasantness rating task in the DRM paradigm, we considered it appropriate to compare the standard version of that task with a supposedly more item-specific version, plus a read-only control condition. Thus, in Experiment 2 we did not include the shallow processing condition and instead focused on the comparison of the two versions mentioned above.

These discrepant results might also be related to certain methodological aspects involved in the DRM paradigm, such as the large number of lists that participants had to study, which could affect the so-called *retention size* (number of lists studied before performing the recognition test, see Jou et al., 2004). Some studies have provided evidence for the claim that when retention size increases, the activation level of the items decreases, weakening verbatim representations and enhancing gist representations, which increases false memories (Gallo, 2004; Jou and Flores, 2013). In our study we tried to match the task demand with previous studies by including 16 DRM lists of 6 associated words. However, the study phase in distinctive encoding research usually consists of only 10-word lists (e.g., Huff and Bodner, 2013, 2019; Gretz and Huff, 2020). Since the number of lists (themes) studied may affect recognition rate, we propose to mitigate this effect by reducing the number of lists studied, but trying to match the task demand in the recognition test. Therefore, in Experiment 2 the number of lists used was reduced from 16 to 12, and the total number of studied words was 72 instead of 96.

## 3 Experiment 2

Experiment 2 focused on the possible influence of distinctive/item-specific processing on false recognition, minimizing the possible effect of retention size. In addition to using a read-only control condition, this time two versions of the pleasantness rating task were compared: the standard version used in Experiment 1 and a supposedly more item-specific version, similar to one of those used by Huff and Bodner (2013). All this provided a more rigorous test of the mirror effect on recognition memory produced by the pleasantness rating in the DRM paradigm.

It was expected to find, as in Experiment 1, that the study of DRM lists would generate a high rate of the false recognition of critical lures, regardless of the type of encoding (classic DRM effect). Importantly, distinctive/specific processing involving pleasantness ratings (in both versions) was predicted to produce higher correct recognition and lower false recognition than the control condition (mirror effect), although the effect would be expected to be greater in the supposedly more specific version.

Based also on the results of Experiment 1, it was predicted that distinctive encoding (in both versions of pleasantness rating), compared to the control condition, would produce higher estimate of recollection in correct recognition. Regarding false recognition, one would expect a less recollection-based experience. Also, the reduction of the DRM illusion when performing distinctive encoding would be due to both encoding and retrieval mechanisms reflected in the SDT analyses.

### 3.1 Materials and methods

#### 3.1.1 Participants

A further 144 psychology students from the University of Santiago de Compostela and the University of Salamanca, all native Spanish speakers, participated voluntarily and received course credit. Data from eight participants were eliminated due to extreme scores, leaving 136 participants for analysis (119 females; *M_age_* = 18.87, *SD* = 2.75). The sample was divided into three groups according to the type of encoding: Control (*n* = 45), Standard (*n* = 46) and Specific (*n* = 45); and the sample size was sufficiently power (0.80) to detect medium-sized effects (*f* = 0.27; Faul et al., 2007).

#### 3.1.2 Design

The only modifications in Experiment 2 were the inclusion of a new level in the Encoding Type variable, the more specific version of the pleasantness rating task, and the elimination of shallow encoding.

#### 3.1.3 Materials

The same materials were used as in Experiment 1, with the following modifications. We tried to control the valence and arousal of the list sets to avoid their effect in false recognition (Chang et al., 2021), so we used the web search engine EmoFinder (Fraga et al., 2018) as a means of finding the valence and arousal values of the words in the lists used. Subsequently, in order to reduce the number of lists, the two lists with the most extreme levels of valence and arousal in each of the sets were eliminated. After pairing the two sets again in terms of BAS, false recognition, arousal and valence, 24 word lists were used for the study phase.

The recognition test was adapted to the new number of word lists. In this case it included 72 words (36 studied, 36 non-studied). The non-studied words corresponded to the 12 critical lures associated with the studied lists, and the rest derived from 6 lists of the non-studied set, 18 unrelated distractor words, and 6 unrelated distractor-critical words. In addition, with the same procedure as in Experiment 1, 4 test versions were created for each set.

#### 3.1.4 Procedure

The only differences from the procedure of Experiment 1 were that each participant studied 12 word lists and that participants in the new study condition were given instructions using the specific version of the pleasantness rating used by Huff and Bodner (2013). They were asked to “think of a single reason why each word is pleasant or not.” In addition, they were requested to judge each word as pleasant or not by pressing the YES or NO buttons on the keyboard.

### 3.2 Results

**Figure 2:**
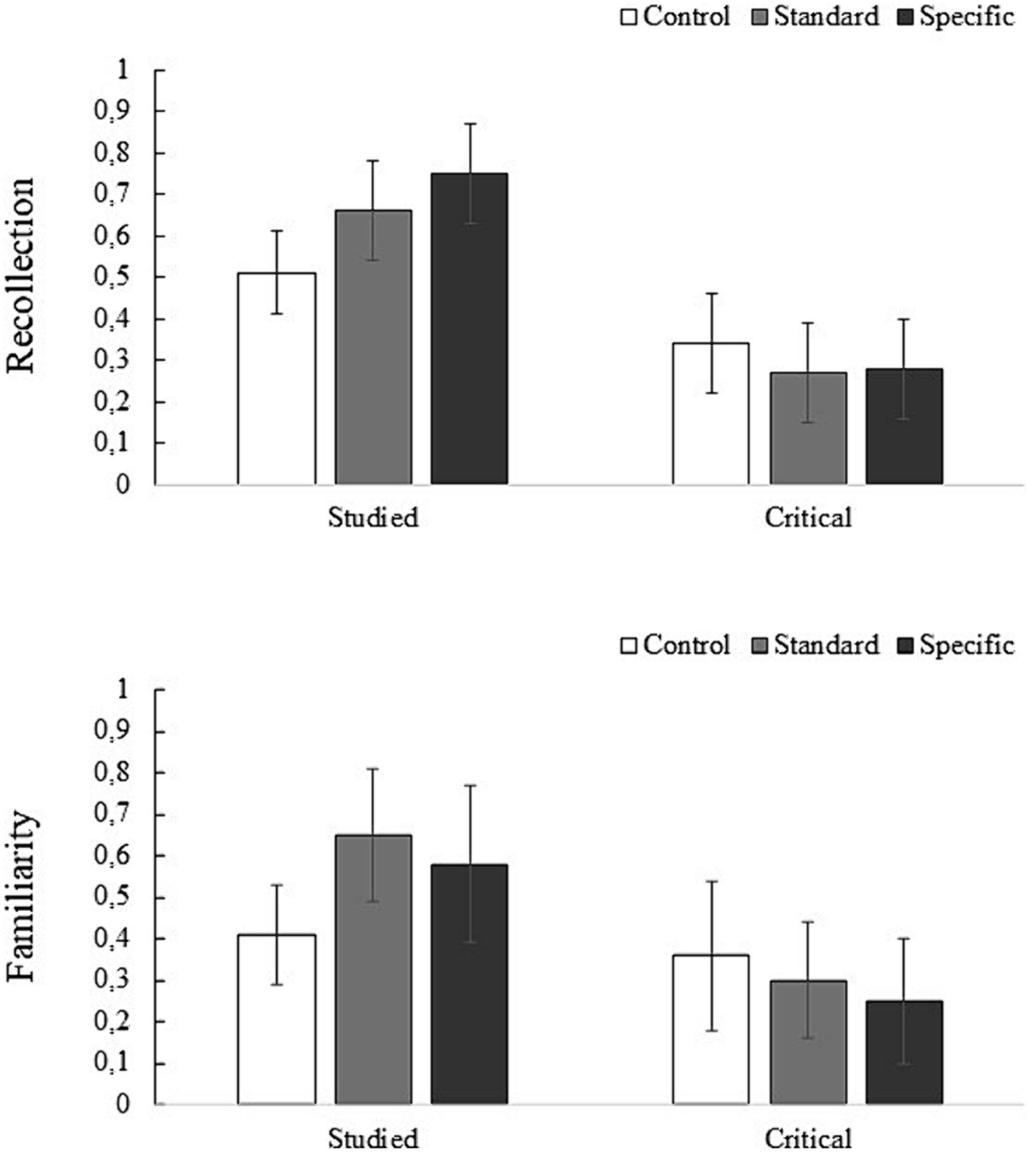
Mean (bars = ± 95% CI) estimates of recollection and familiarity for studied words and critical lures by encoding type for Experiment 2.

The same analyses were carried out as in Experiment 1. Table 2 sets out the mean proportion of “Yes” responses and mean signal-detection indexes on the recognition test as a function of item type for the encoding conditions. Figure 2 shows the recollection and familiarity estimates for the studied and critical items for each type of encoding.

**Table 2:** Mean (*SD*) proportion of “yes” responses and signal-detection indexes on the recognition test as a function of encoding condition and item type for Experiment 2.

Encoding group/item type/index	Control	Standard	Specific
*n*	45	46	45
List items	0.75 (0.11)	0.92 (0.08)	0.92 (0.07)
Unrelated distractors	0.07 (0.07)	0.06 (0.06)	0.04 (0.05)
List-items *d’*	2.23 (0.50)	3.11 (0.62)	3.24 (0.60)
List-items *λ*	1.45 (0.44)	1.50 (0.42)	1.57 (0.43)
Critical lures	0.61 (0.22)	0.52 (0.21)	0.48 (0.21)
Unrelated-critical distractors	0.06 (0.08)	0.05 (0.08)	0.04 (0.07)
Critical-lures *d’*	1.57 (0.68)	1.34 (0.66)	1.21 (0.64)
Critical-lures *λ*	1.50 (0.39)	1.55 (0.36)	1.68 (0.29)

#### 3.2.1 Correct recognition

##### 3.2.1.1 Accuracy

As in Experiment 1, correct recognition differed by the encoding type, *F*(2, 133) = 57.07, *p* < 0.001, *η*_p_^2^ = 0.46. Although no significant differences were observed between the standard and specific conditions (0.92 vs. 0.93), *t*(89) = 0.14, *p* = 1.000, correct recognition was greater in both than in the control condition (0.92 vs. 0.75), *t*(89) = 9.22, *p* < 0.001, and (0.93 vs. 0.75), *t*(88) = 9.31, *p* < 0.001, respectively. The ANOVA performed on the *d’*_list_ index showed a significant effect for the type of encoding, *F*(2, 133) = 40.84, *p* < 0.001, *η*_p_^2^ = 0.38. Memory information of studied words was equivalent for the standard and specific conditions (3.11 vs. 3.24), *t*(89) = 1.06, *p* = 0.878; however, both of these obtained a greater *d’*_list_ compared to control condition (3.11 vs. 2.23), *t*(89) = 7.28, *p* < 0.001, and (3.24 vs. 2.23), *t*(88) = 8.29, *p* < 0.001, respectively. Finally, no significant effects were found for the type of encoding in the analysis of the monitoring of studied words, index *λ*_list_, *F*(2, 133) = 3.03, *p* = 0.052.

##### 3.2.1.2 Subjective retrieval experience

The ANOVA performed on the recollection estimate of the studied words showed a significant effect of encoding type, *F*(2, 133) = 19.51, *p* < 0.001, *η*_p_^2^ = 0.23. The standard and specific conditions showed higher recollection than in the control condition (0.67 vs. 0.51), *t*(89) = 4.07, *p* < 0.001, and (0.75 vs. 0.51), *t*(88) = 6.15, *p* < 0.001, respectively. Nevertheless, the recollection was equivalent for the standard and specific conditions (0.67 vs. 0.75), *t*(89) = 2.16, *p* = 0.098. In addition, the same ANOVA on the familiarity estimate of the studied words showed an effect for encoding type, *F*(2, 132) = 9.61, *p* < 0.001, *η*_p_^2^ = 0.13. The standard and specific conditions showed higher familiarity than in the control condition (0.65 vs. 0.41), *t*(89) = 4.29, *p* < 0.001, and (0.58 vs. 0.41), *t*(87) = 2.91, *p* = 0.013, respectively. However, the familiarity was equivalent for the standard and specific conditions (0.65 vs. 0.58), *t*(88) = 1.34, *p* = 0.545.

#### 3.2.2 False recognition

##### 3.2.2.1 Accuracy

The ANOVA performed on the recognition of the critical lures showed a significant effect of encoding type, *F*(2, 133) = 4.49, *p* = 0.013, *η*_p_^2^ = 0.06. The standard condition showed no difference in false recognition with either the control condition, (0.52 vs. 0.61), *t*(89) = 2.01, *p* = 0.141, or the specific condition, (0.52 vs. 0.48), *t*(89) = 0.94, *p* = 1.000; however, false recognition was lower in the specific condition than in the control one, (0.48 vs. 0.61), *t*(88) = 2.93, *p* = 0.012. The same ANOVA on *d’*_critical_ showed a significant effect for type of encoding, *F*(2, 133) = 3.31, *p* = 0.040, *η*_p_^2^ = 0.05. Memory information of critical lures was equivalent for the standard and control conditions, (1.34 vs. 1.57), *t*(89) = 1.61, *p* = 0.328, and, also, standard and specific conditions, (1.34. vs. 1.21), *t*(89) = 0.94, *p* = 1.000. Importantly, specific condition obtained a lower *d’*_critical_ compared to control condition (1.21 vs. 2.23), *t*(89) = 7.28, *p* < 0.001. Furthermore, no significant effects were found for the type of encoding in the analysis of variance of the index *λ*_critical_, *F*(2, 133) = 0.75, *p* = 0.472.

##### 3.2.2.2 Subjective retrieval experience

In addition, an ANOVA conducted on the recollection estimate of the critical lures showed no significant effect of encoding type, *F*(2, 133) = 1.01, *p* = 0.368. Again, the ANOVA on the familiarity estimate of the critical lures showed no effect of encoding type, *F*(2, 133) = 2.65, *p* = 0.074.

### 3.3 Discussion

In line with Experiment 1, in the conditions where deep/distinctive encoding is performed (the two versions of the pleasantness rating task), higher correct recognition was achieved compared to the control condition (reading only). Moreover, there were no differences between the two pleasantness rating tasks, thus they appear to be equally effective in enhancing recognition performance. This is shown by the greater amount of encoded information in both conditions compared to the control (*d’*_list_). However, the two pleasantness rating tasks showed no significant differences in monitoring (*λ*_list_) relative to the control condition.

In terms of the DRM illusion, as in Experiment 1, false recognition was similar between standard and control conditions. In contrast, we observed that the specific condition reduced false recognition relative to the control condition, showing on this occasion the expected mirror effect of item-specific processing in recognition (e.g., Huff and Bodner, 2013). We further showed that the specific condition resulted in less encoded information (*d’*_critical_), whereas the standard one did not. However, we found that the specific and standard condition did not increase monitoring (*λ*_critical_) at test relative to the control. We will address this pattern in more detail in the general discussion, but we note that the reduced number of lists studied by the participants did not allow the standard task to produce the mirror effect.

Regarding the subjective retrieval experience, we observed differences based on the type of encoding only in correct recognition. The two pleasantness rating tasks showed a higher estimate of recollection and familiarity in the studied items relative to the control condition. The typical effect of processing levels (Gardiner and Parkin, 1990) on the two tasks that perform deep/distinctive processing is again apparent. In terms of false recognition, contrary to expectations, the subjective experience of retrieval does not differ by type of encoding. Although the specific condition succeeded in reducing false recognition, no reduction in recollection-based recognition, i.e., the experience of retrieving distinctive, but illusory, details about critical lures, was observed. This is probably a consequence of ineffective monitoring at test.

## 4 General discussion

The main objective of our research was to explore the issue of under what conditions the pleasantness rating task can produce the mirror effect in the DRM paradigm, based on evidence of recognition accuracy and subjective retrieval experience. Across experiments, the standard pleasantness rating task, relative to a read-only control and a shallow processing condition, improved correct recognition, but did not reduce false recognition. Only the specific pleasantness rating task succeeded in producing the expected mirror effect. This pattern of results was accompanied by the subjective retrieval experience, with correct recognition being more based on recollection and familiarity than control, with no differences found in false recognition.

In Experiment 1, the failure of the standard task to produce the mirror effect may have been due to methodological aspects. We know that recognition can be affected by a number of factors, such as the amount of information presented to be studied (Jou et al., 2004; Jou and Flores, 2013). The high number of study lists (themes) could weaken the verbatim representations and increase the gist representations of items. This could generate a condition that prevents the reduction of false recognition by the standard task. Although in Experiment 1 we tried to control for this effect by matching the demand of the task to that of other similar studies (Huff and Bodner, 2013; Gretz and Huff, 2020; Huff et al., 2020, 2021), it is possible that we were not successful in this. Bearing in mind the importance of retention size, in Experiment 2 we considered whether false recognition could be reduced with the standard task by presenting less information to be studied. At the same time, to examine whether the standard task performed item-specific processing in recognition, we used, in addition to the read-only control, a more specific version of the pleasantness rating task to compare with the standard task. If the specific version managed to reduce false recognition and the standard task was incapable of doing this, we would have evidence to believe that processing similar to relational processing is performed by means of the standard instruction.

In Experiment 2, despite the new study conditions (12 lists of 6 words), the standard task showed no reduction in false recognition relative to the control. In contrast, the specific version of the task reduced false recognition only relative to the control, not to the standard task. Also, very importantly, the subjective retrieval experience accompanied the pattern of recognition outcomes. The processes of recollection and familiarity, although performing different actions, influence recognition in a similar way, unlike free recall, where recollection has more influence (Uner and Roediger, 2022). What we have observed here is that, when distinctive encoding is performed, both processes seem to be activated to facilitate the recognition of the studied words. Thus, we found higher recollection and familiarity estimates in the studied words in the two distinctive encoding tasks. Conversely, in the case of critical lures, the subjective retrieval experience is not modified by the type of encoding. When distinctive processing is performed and false recognition is reduced, we expected to find a reduction in the recollection process, but that the familiarity process would remain unchanged. This phenomenon would be related to what occurs with the processes of the inflating and editing of errors (Arndt and Gould, 2006); while both work together to enhance correct memories, in false memories the inflating process increases them (similar to the familiarity process) and the editing process reduces them (similar to the recollection process). However, even the specific task, which reduced false recognition, did not show less recollection-based recognition. The following question arises from these results: Does the DRM illusion reduction effect not appear due to the study conditions, the task instructions, or both?

Regarding the study conditions, in our study we used materials in Spanish (Beato and Díez, 2011), a language that has not been used in previous work here. The particularity of these lists lies in the fact that they contain 6 study items that were simultaneously related to three critical lures, although we only used the critical lure that generated the most false recognition. Whereas the DRM paradigm has typically been studied with lists of 12 associated words (Roediger and McDermott, 1995; see also Gallo, 2010), Beato and Díez’s lists have been used in several studies to explore different effects on the DRM illusion (e.g., Cadavid and Beato, 2016; Pitarque et al., 2018; Beato et al., 2023). On the same lines, we found that the number of associates studied is another variable in the study phase that may affect false recognition. We know that the higher the number of studied associates in the DRM lists, the higher the level of false memories (e.g., Arndt and Gould, 2006). The lists used in our study, containing 6 associated words, generate lower levels of false recognition than lists of 12–15 words. Thus, we considered whether the baseline level of false recognition could affect the reduction of the DRM illusion. Reviewing previous work on the reduction of false recognition, we found that when the same study lists were used, the reduction of the DRM illusion occurs when using other strategies, such as the recall-to-reject strategy based on feelings of contrast (e.g., Cadavid et al., 2021). However, we do not know whether a low false recognition baseline level can affect the reduction of the DRM illusion when performing a distinctive encoding and, specifically, when performing a pleasantness rating task. Previous research has only found the mirror effect at high baseline levels of false recognition (e.g., Huff and Bodner, 2013; Huff et al., 2020).

Concerning the instruction of the pleasantness rating task, we know that the benefits of distinctiveness encoding occur through the encoding and retrieval processes (Huff et al., 2015). Performing item-specific processing leads to impoverished relational encoding and enhanced test monitoring through the distinctiveness heuristic, and consequently to better memory performance (e.g., Huff and Bodner, 2013, 2019; Huff and Aschenbrenner, 2018). However, in our study this pattern has not been reflected in signal detection analyses. The pleasantness rating tasks involved the accumulation of a large amount of information about the studied items, and thus result in better correct recognition, but only the specific task reduced the information about the critical lures. Moreover, focusing on the retrieval process, the pleasantness rating tasks did not lead to appropriate monitoring, even on correct recognition. In fact, the reduction in false recognition by the specific task is mainly due to impoverished relational processing and not to increased test monitoring. Therefore, we detected a generalized failure in the monitoring strategy. This failure is further evidenced by the subjective retrieval experience. One of the consequences of not performing adequate monitoring is that the encoded information about the studied words is misattributed to critical lures at test (Gallo, 2004). The specific task continued to base its false recognition on the recollection process in the same way as the control and standard conditions, i.e., it continued to retrieve specific details about the critical lures despite reducing false recognition.

One possible explanation for not finding the mirror effect with the standard pleasantness rating task in these study conditions could be that the instructions in this task do not induce sufficiently item-specific processing. Such an explanation would be justified for several reasons. When comparing the standard version of the pleasantness rating task with the versions designed by Huff and Bodner (2013), we see that it presents instructions that are more similar to the relational version. In both the standard version and the relational version, pleasantness is assessed on a Likert-type scale; the difference is that in the standard version the items do not have to be compared with each other. Regarding the item-specific version, they are asked to think of a single reason why each word is or is not pleasant. This instruction, in terms of encoding, encourages the search for differences between the words in the list, while rating the pleasantness of each item on a scale does not allow for working with different information between the items on a list. It is probable that the words of the same list share a similar subjective pleasantness, since they are semantically related, and participants thus continue to work indirectly with relational information. This effect could be predicted from the assumptions of the fuzzy-trace theory (Brainerd and Reyna, 2002). If the level of pleasantness is similar across list items, this may promote a more robust gist trace and/or not promote a verbatim trace. That is, error-inflating processes could be increased but not error-editing processes, resulting both higher correct and false recognition.

The fact that, in contrast to other studies that used these same versions of the pleasantness rating task, we detected a failure in the monitoring strategy for both tasks, suggests that the study conditions may be interacting with the type of processing in the encoding. We know that some study conditions can generate costs and spillover effects, for example, the distinctive encoding of a subset of DRM lists (Huff et al., 2021). This phenomenon may have taken place in the study by Gretz and Huff (2020), where the mirror effect on recognition was not found using the standard version of the task. Our study, on the contrary, differs from previous research in the use of DRM lists with fewer words, which resulted in a lower baseline level of false recognition. Here we propose the baseline level of false recognition as a possible boundary condition. Perhaps when there are circumstances in which false recognition is very high, and therefore relational processing is very intense, the standard pleasantness rating task may help to reduce false recognition. On the other hand, in circumstances of lower false recognition it may not be as effective as other more item-specific tasks. It is also worth mentioning that the task instructions do not lead to pure processing, either relational or specific, but rather a bias in the type of processing to be performed (Jacoby, 1991; Huff and Bodner, 2013; Huff et al., 2021). Thus, some instructions are more successful in inducing distinctive processing than others. More research is needed regarding the effects of distinctive encoding on the pleasantness rating task; for example, future studies might consider whether the baseline level of false recognition is a boundary condition in this task and not in others.

The study of the characterization of the subjective experience associated with false memories continues to be a topic of great interest. It has been investigated with different populations (e.g., Prull et al., 2006; Caza et al., 2011), by manipulating encoding (e.g., Pérez-Mata et al., 2022), manipulating retrieval (e.g., Uner and Roediger, 2022) and combining behavioral and electrophysiological measures (e.g., Boldini et al., 2013; Dimsdale-Zucker et al., 2022). However, there are still areas to be resolved (Schacter et al., 2021). We believe that the subjective retrieval experience is a perfect ally for the study of the reduction of false memories through distinctive encoding. At the same time, we emphasize that signal detection analyses can help to separate the effects of encoding from those of retrieval in different types of manipulations (e.g., Nieznański et al., 2018). Using quantitative measures, such as accuracy and indexes of discriminability and response bias, and with qualitative measures, such as the subjective retrieval experience, great strides can be made in understanding memory illusions.

## 5 Conclusion

Our results highlight the ability of the standard pleasantness rating task to increase correct recognition while not reducing false recognition in these study conditions. This task seems to differ from the specific task, which does achieve the mirror effect. We consider that the standard task does not perform sufficiently item-specific processing. However, we also consider the baseline level of false recognition a boundary condition for research on distinctive encoding. Finally, we consider not only recognition accuracy but also the subjective retrieval experience to be essential measures in the study of false memories.

## Data availability statement

The datasets presented in this study can be found in online repositories. The names of the repository/repositories and accession number(s) can be found at: Participant data for all experiments reported are available via our OSF project: https://osf.io/rzyjc/.

## Ethics statement

The studies involving humans were approved by the Bioethics Committee of the University of Santiago de Compostela. The studies were conducted in accordance with the local legislation and institutional requirements. The participants provided their written informed consent to participate in this study.

## Author contributions

AA-M: Data curation, Investigation, Methodology, Software, Writing – original draft, Writing – review & editing. MS-V: Conceptualization, Writing – review & editing. JF-R: Conceptualization, Supervision, Writing – review & editing, Writing – original draft.

## Footnotes

^1^We are aware that a large part of the student body at the University of Santiago de Compostela is fluent in Spanish and Galician. This was not considered a problem since false memories do not differ between two languages when speakers have the same proficiency in both (Suarez and Beato, 2021).

^2^z-scores were calculated using the inverse of the normal distribution function (Hautus et al., 2021).
