# Supplementary material for: Examining the effects of pleasantness ratings on correct and false recognition in the DRM paradigm: accuracy, recollection and familiarity estimates
Source: Front Psychol. 2024 Mar 20;15:1265291. doi: 10.3389/fpsyg.2024.1265291 (PMC10988982; doi:10.3389/fpsyg.2024.1265291)
Supplement: Supplementary file 1 [file Data_Sheet_1.DOCX]

Supplementary Material

Examining the effects of pleasantness ratings on correct and false recognition in the DRM paradigm: accuracy, recollection and familiarity estimates.

Alicia Alvarez-Martinez, Maria J. Sampedro-Vizcaya, Jose Fernandez-Rey*

*** Correspondence:** jose.fernandez.rey@usc.es

# Supplementary Data

## Set of DRM lists used in the study

The backward associative strength (BAS) value of each critical word with its list and the average percentage of false recognition of the critical word are included. Data obtained from the studies of Beato and Cadavid (2016) and Beato and Díez (2011). In addition, the mean valence and arousal value of the words in each list is shown, excluding the critical word, information obtained from EmoFinder (Fraga et al., 2018).

| **Set 1 lists** | **MValence** | **MArousal** | **BAS** | **FR** |
| --- | --- | --- | --- | --- |
| monedero (purse)  cobrar (to charge)  salario (salary)  empleo (employment)  jornal (day´s pay)  paga (pay)  TRABAJO (WORK) | 6,76 | 6,28 | 0,15 | 64,81 |
| daño (damage)  rasguño (scratch)  corte (cut)  pinchazo (prick)  ampolla (blister)  cicatriz (scar)  HERIDA (INJURY) | 3,06 | 6,52 | 0,89 | 61,11 |
| nupcial (bridal)  novios (newlyweds)  enlace (bond)  compromiso (commitment)  pareja (couple)  casar (to marry)  MATRIMONIO (MARRIAGE) | 6,54 | 6,07 | 0,30 | 75,86 |
| balón (ball)  deportes (sports)  béisbol (baseball)  baloncesto (basketball)  bola (pellet)  tenis (tennis)  PELOTA (GOLF OR TENNIS BALL) | 5,73 | 5,44 | 0,15 | 51,72 |
| guateque (bash)  verbena (open-air dance)  festival (festival)  baile (dance)  concierto (concert)  discoteca (disco)  FIESTA (PARTY) | 7,13 | 6,56 | 0,83 | 51,72 |
| explosión (explosion)  cañón (cannon)  misil (missile)  dinamita (dynamite)  destrucción (destruction)  barricada (barricade)  BOMBA (BOMB) | 3,16 | 7,08 | 0,25 | 62,07 |
| rejas (bars)  prisionero (prisioner)  celda (cell)  reo (offender)  presidio (penitentiary)  reclusión (imprisonment)  PRISIÓN (PRISON) | 2,83 | 6,31 | 0,19 | 84,38 |
| cenicero (ashtray)  pipa (pipe)  humo (smoke)  mechero (lighter)  puro (cigar)  pulmones (lungs)  FUMAR (TO SMOKE) | 4,73 | 4,92 | 0,64 | 31,25 |
| bufanda (scarf)  manta (blanket)  invierno (winter)  jersey (jersey)  escalofrío (chill)  gabardina (raincoat)  ABRIGO (COAT) | 5,73 | 4,27 | 0,20 | 40,63 |
| fallecimiento (demise)  pésame (condolence)  entierro (burial)  pena (sorrow)  agonía (agony)  funeral (funeral)  MUERTE (DEATH) | 1,81 | 6,98 | 0,87 | 34,38 |
| navegación (navigation)  puerto (port)  navío (ship)  marinero (sailor)  flota (fleet)  océano (ocean)  BARCO (BOAT) | 6,33 | 4,61 | 0,62 | 46,83 |
| modista (dressmaker)  percha (hanger)  vestir (to dress)  tela (cloth)  elegante (elegant)  tejido (fabric)  VESTIDO (DRESS) | 5,86 | 4,35 | 0,16 | 55,17 |
| maullido (miaow)  mascota (pet)  maullar (to meow)  pulgas (fleas)  rabo (tail)  veterinaria (veterinary)  GATO (CAT) | 6,01 | 5,51 | 0,76 | 24,14 |
| jazmín (jasmine)  perfume (perfume)  aroma (aroma)  fragancia (fragance)  esencia (essence)  violeta (violet)  COLONIA (COLOGNE) | 6,92 | 4,15 | 0,53 | 31,03 |
| día (day)  luna (moon)  amanecer (dawn)  sombra (shadow)  estrella (star)  atardecer (dusk)  NOCHE (NIGHT) | 6,90 | 3,91 | 0,24 | 41,38 |
| vivienda (dwelling)  portal (doorway)  fachada (facade)  arquitecto (architect)  ático (attic)  viga (beam)  EDIFICIO (BUILDING) | 5,93 | 4,64 | 0,42 | 44,83 |
| **Set 2 lists** | **Valencia** | **Arousal** | **BAS** | **FR** |
| batido (milk shake)  sabor (flavor)  caramelo (candy)  pastel (pie)  mermelada (jam)  tarta (cake)  DULCE (SWEET) | 7,06 | 4,55 | 0,87 | 33,33 |
| contagio (contagion)  virus (virus)  constipado (to have a cold)  tos (cough)  resfriado (cold)  estornudo (sneeze)  CATARRO (CATARRH) | 2,80 | 6,57 | 0,17 | 66,67 |
| óptica (optics)  ojo (eye)  lentillas (contact lenses)  prismáticos (binoculars)  lupas (magnifiers)  visión (visión)  GAFAS (GLASSES) | 5,81 | 4,42 | 0,06 | 51,85 |
| villa (town)  municipio (municipality)  localidad (locality)  comarca (district)  localización (location)  región (región)  CIUDAD (CITY) | 5,44 | 4,53 | 0,23 | 37,93 |
| poderoso (powerful)  palacio (palace)  aristocracia (aristocracy)  lujo (luxury)  nobleza (nobility)  poder (power)  RIQUEZA (WEALTH) | 5,81 | 5,73 | 0,19 | 44,83 |
| glaciar (glacier)  Antártida (Antarctica)  iceberg (iceberg)  pingüino (penguin)  iglú (igloo)  esquimal (eskimo)  HIELO (ICE) | 5,85 | 4,78 | 0,81 | 24,14 |
| clérigo (cleric)  sotana (cassock)  sacerdote (priest)  fraile (friar)  monasterio (monastery)  monja (nun)  MONJE (MONK) | 4,41 | 4,20 | 0,08 | 68,97 |
| cauce (riverbed)  pez (fish)  orilla (shore)  lago (lake)  barca (boat)  bahía (bay)  RÍO (RIVER) | 6,22 | 3,56 | 0,59 | 51,72 |
| mili (military service)  coronel (colonel)  cuartel (barracks)  infantería (infantry)  general (general)  legión (legion)  MILITAR (MILITARY) | 4,25 | 5,68 | 0,28 | 62,07 |
| cama (bed)  pesadilla (nightmare)  camisón (nightdress)  descansar (to rest)  soñar (to dream)  cansancio (tiredness)  SUEÑO (DREAM) | 5,64 | 3,91 | 0,42 | 82,76 |
| vodka (vodka)  juerga (spree)  ron (rum)  licor (liqueur)  borracho (drunk)  borrachera (drunkenness)  ALCOHOL (ACOHOL) | 5,10 | 6,35 | 0,72 | 34,38 |
| terror (terror)  pavor (dread)  horror (horror)  aterrorizado (terrified)  susto (scare)  temeroso (fearful)  TEMOR (AWE) | 2,56 | 7,19 | 0,03 | 75,00 |
| gel (gel)  toalla (towel)  bañera (bath)  servicio (toilet)  lavabo (sink)  jabón (soap)  BAÑO (BATHROOM) | 5,74 | 3,94 | 0,49 | 37,93 |
| escena (scene)  interpretación (interpretation)  escenario (stage)  actriz (actress)  intérprete (interpreter)  actuar (to act)  TEATRO (THEATRE) | 6,15 | 5,61 | 0,77 | 48,28 |
| balas (bullets)  disparo (shot)  revólver (revolver)  rifle (rifle)  fusil (handgun)  metralleta (machine gun)  PISTOLA (PISTOL) | 2,67 | 7,12 | 0,23 | 43,83 |
| carcajada (laugh)  humor (humor)  sonrisa (smile)  gracia (jocularity)  diversión (fun)  simpatía (sympathy)  RISA (LAUGHTER) | 8,16 | 5,75 | 0,27 | 65,52 |

Note: For experiment 2 the eliminated lists were those corresponding to the critical words: Prison, Party, Death, Cologne, Sweet, Laughter, Pistol and Awe. In addition, the Cat and Night lists were moved to set 2 and the Wealth and Ice lists were moved to set 1.

## Raw Remember/Know responses

### TABLE 1. Mean (SD) proportion of yes, remember and know responses as a function of type of encoding and item type for Experiment 1.

| **Type of encoding** | **Type of item** | **Proportion of yes response** | | |
| --- | --- | --- | --- | --- |
|  |  | **Total** | **Remember** | **Know** |
| Control | Studied | .68 (.12) | .47 (.15) | .21 (.10) |
|  | Critical | .46 (.23) | .22 (.18) | .24 (.17) |
|  | Distractor | .05 (.07) | .01 (.02) | .04 (.06) |
|  | Distractor-critical | .12 (.15) | .03 (.07) | .09(.13) |
| Standard | Studied | .88 (.10) | .65 (.25) | .24 (.25) |
|  | Critical | .44 (.19) | .27 (.16) | .17 (.14) |
|  | Distractor | .05 (.07) | .01 (.04) | .04 (.05) |
|  | Distractor-critical | .11 (.14) | .03 (.07) | .08 (.12) |
| Shallow | Studied | .52 (.14) | .31 (.16) | .20 (.07) |
|  | Critical | .43 (.16) | .17 (.12) | .25 (.12) |
|  | Distractor | .14 (.11) | .05 (.07) | .08 (.08) |
|  | Distractor-critical | .21 (.19) | .06 (.12) | .15 (.16) |

### TABLE 2. Mean (SD) proportion of yes, remember and know responses as a function of type of encoding and item type for Experiment 2.

| **Type of encoding** | **Type of item** | **Proportion of yes response** | | |
| --- | --- | --- | --- | --- |
|  |  | **Total** | **Remember** | **Know** |
| Control | Studied | .72 (.13) | .48 (.19) | .24 (.14) |
|  | Critical | .59 (.22) | .34 (.20) | .28 (.19) |
|  | Distractor | .08 (.09) | .03 (.05) | .05 (.07) |
|  | Distractor-critical | .12 (.16) | .04 (.10) | .09 (.14) |
| Standard | Studied | .93 (.08) | .64 (.21) | .28 (.19) |
|  | Critical | .54 (.22) | .28 (.22) | .25 (.16) |
|  | Distractor | .07 (.08) | .02 (.04) | .05 (.08) |
|  | Distractor-critical | .09 (.13) | .03 (.08) | .06 (.10) |
| Specific | Studied | .93 (.06) | .76 (.19) | .17 (.18) |
|  | Critical | .52 (.22) | .33 (.20) | .19 (.15) |
|  | Distractor | .07 (.09) | .02 (.04) | .04 (.08) |
|  | Distractor-critical | .09 (.13) | .04 (.09) | .04 (.09) |
